# Draft Genome Sequence of the Nylon Oligomer-Degrading Bacterium *Arthrobacter* sp. Strain KI72

**DOI:** 10.1128/genomeA.00217-17

**Published:** 2017-04-27

**Authors:** Ikki Takehara, Dai-Ichiro Kato, Masahiro Takeo, Seiji Negoro

**Affiliations:** a Department of Applied Chemistry, Graduate School of Engineering, University of Hyogo, Himeji, Hyogo, Japan; b Graduate School of Science and Engineering, Kagoshima University, Korimoto, Kagoshima, Japan

## Abstract

We report here the 4.6-Mb genome sequence of a nylon oligomer-degrading bacterium, *Arthrobacter* sp. strain KI72. The draft genome sequence of strain KI72 consists of 4,568,574 bp, with a G+C content of 63.47%, 4,372 coding sequences (CDSs), 54 tRNAs, and six rRNAs.

## GENOME ANNOUNCEMENT

The genus *Arthrobacter* was first proposed as *Arthrobacter globiformis* by Conn and Dimmick (1). *Arthrobacter* sp. strain KI72 is a Gram-positive aerobic rod bacterium which is able to grow on 6-aminohexanoic acid (Ahx) oligomer (designated nylon oligomer, a by-product of nylon-6 manufacture) as the sole source of carbon and nitrogen. With regard to the enzymatic system responsible for the metabolism of the unnatural amide compounds, we have been studying the degradation of Ahx oligomers by strain KI72 as the model system (2–10). The strain KI72 harbors three kinds of plasmids, pOAD1 (39.7 kbp), pOAD2 (43.6 kbp), and pOAD3 (56.3 kbp). Previous biochemical studies have revealed that three enzymes encoded on pOAD2, NylABC, are responsible for the degradation of Ahx oligomers to monomer form (2–6). The Ahx cyclic dimer hydrolase (NylA; EC 3.5.2.12), a member of the amidase signature hydrolase family, specifically hydrolyzes one of the two amide bonds in the Ahx cyclic dimer, generating an Ahx linear dimer (7). The Ahx dimer hydrolase (NylB; EC 3.5.1.46), a member of the penicillin-recognizing family of serine reactive hydrolases, hydrolyzes Ahx oligomers by an exo-type mode (8, 9). The Ahx oligomer hydrolase (NylC; EC 3.5.-.-) degrades Ahx cyclic and linear oligomers with a degree of polymerization greater than three by an endo-type mode (10). Moreover, we have revealed the three-dimensional structures of NylABC by X-ray crystallographic analyses (7–10). Recently, we found that a thermostable NylC mutant enzyme degrades various aliphatic nylons (11). Here, we present the draft genome sequence of *Arthrobacter* sp. strain KI72 to better understand the metabolism of nylons and their related compounds.

The genome of strain KI72 was sequenced using a shotgun reads obtained on a HiSeq sequencer (Illumina). A total of 78,562,074 reads with an average length of 50.00 nucleotides (~858.3-fold coverage) were used for genome assembly by the Edena assembler version 3 (Genomic Research Laboratory) (12). A total of 105 contigs of >100 bp in length were constructed, with an *N*_50_ of 143.01 kb; the largest contig assembled measured 372.75 kbp. The final draft genome sequence consists of 4,568,574 bp, and Microbial Genome Annotation Pipeline (MiGAP) was used for genome annotation (13). The draft genome of strain KI72 contains 4,372 coding sequences (CDSs), 54 tRNAs, and six rRNAs, and the G+C content was 63.47%.

The complete annotated genome sequence shows that strain KI72 contains genes encoding a novel NylC-like amidehydrolase. Also, we find the genes which encode 4-aminobutyrate aminotransferase, succinate semialdehyde dehydrogenase, acyl-coenzyme A (acyl-CoA) dehydrogenase, enoyl-CoA hydratase, 3-hydroxyacyl-CoA dehydrogenase, and acetyl-CoA acyltransferase, which are predicted to be relevant to the subsequent degradation of nylon monomer.

### Accession number(s).

This whole-genome shotgun project has been deposited at DDBJ/EMBL/Genbank under the accession number BDMH00000000.

## References

[1] Conn HJ, Dimmick I 1947 Soil bacteria similar in morphology to *Mycobacterium* and *Corynebacterium*. J Bacteriol 54:291–303. 16561362 10.1128/jb.54.3.291-303.1947 PMC526554

[2] Negoro S 2000 Biodegradation of nylon oligomers. Appl Microbiol Biotechnol 54:461–466. doi:10.1007/s002530000434 11092619

[3] Okada H, Negoro S, Kimura H, Nakamura S 1983 Evolutionary adaptation of plasmid-encoded enzymes for degrading nylon oligomers. Nature 306:203–206. doi:10.1038/306203a0 6646204

[4] Kato K, Ohtsuki K, Koda Y, Maekawa T, Yomo T, Negoro S, Urabe I 1995 A plasmid encoding enzymes for nylon oligomer degradation: nucleotide sequence and analysis of pOAD2. Microbiology 141:2585–2590. doi:10.1099/13500872-141-10-2585 7582019

[5] Yasuhira K, Uedo Y, Takeo M, Kato D, Negoro S 2007 Genetic organization of nylon-oligomer-degrading enzymes from an alkalophilic bacterium *Agromyces* sp. KY5R. J Biosci Bioeng 104:521–524. doi:10.1263/jbb.104.521 18215642

[6] Yasuhira K, Tanaka Y, Shibata H, Kawashima Y, Ohara A, Kato D, Takeo M, Negoro S 2007 6-Aminohexanoate oligomer hydrolases from the alkalophilic bacteria *Agromyces* sp. strain KY5R and *Kocuria* sp. strain KY2. Appl Environ Microbiol 73:7099–7102. doi:10.1128/AEM.00777-07 17827307 PMC2074972

[7] Yasuhira K, Shibata N, Mongami G, Uedo Y, Atsumi Y, Kawashima Y, Hibino A, Tanaka Y, Lee YH, Kato D, Takeo M, Higuchi Y, Negoro S 2010 X-ray crystallographic analysis of the 6-aminohexanoate cyclic dimer hydrolase: catalytic mechanism and evolution of an enzyme responsible for nylon-6 byproduct degradation. J Biol Chem 285:1239–1248. doi:10.1074/jbc.M109.041285 19889645 PMC2801252

[8] Negoro S, Ohki T, Shibata N, Mizuno N, Wakitani Y, Tsurukame J, Matsumoto K, Kawamoto I, Takeo M, Higuchi Y 2005 X-ray crystallographic analysis of 6-aminohexanoate-dimer hydrolase: molecular basis for the birth of a nylon oligomer degrading enzyme. J Biol Chem 280:39644–39652. doi:10.1074/jbc.M505946200 16162506

[9] Negoro S, Ohki T, Shibata N, Sasa K, Hayashi H, Nakano H, Yasuhira K, Kato D, Takeo M, Higuchi Y 2007 Nylon-oligomer degrading enzyme-substrate complex: catalytic mechanism of 6-aminohexanoate-dimer hydrolase. J Mol Biol 370:142–156. doi:10.1016/j.jmb.2007.04.043. 17512009

[10] Negoro S, Shibata N, Tanaka Y, Yasuhira K, Shibata H, Hashimoto H, Lee YH, Oshima S, Santa R, Oshima S, Mochiji K, Goto Y, Ikegami T, Nagai K, Kato D, Takeo M, Higuchi Y 2012 Three-dimensional structure of nylon hydrolase and mechanism of nylon-6 hydrolysis. J Biol Chem 287:5079–5090. doi:10.1074/jbc.M111.321992 22187439 PMC3281642

[11] Nagai K, Iida K, Shimizu K, Kinugasa R, Izumi M, Kato D, Takeo M, Mochiji K, Negoro S 2014 Enzymatic hydrolysis of nylons: quantification of the reaction rate of nylon hydrolase for thin-layered nylons. Appl Microbiol Biotechnol. 98:8751–8761. doi:10.1007/s00253-014-5885-2 24962117

[12] Hernandez D, François P, Farinelli L, Osterås M, Schrenzel J 2008 *De novo* bacterial genome sequencing: millions of very short reads assembled on a desktop computer. Genome Res 18:802–809. doi:10.1101/gr.072033.107. 18332092 PMC2336802

[13] Sugawara H, Ohyama A, Mori H, Kurokawa K 2009 Microbial Genome Annotation Pipeline (MiGAP) for diverse users, abstr S001-1-2 *In* Abstr 20th Int Conf Genome Informatics, 14 to 16 December 2009, Kanagawa, Japan.

